# Masticatory Performance Test Using a Gummy Jelly for Older People with Low Masticatory Ability

**DOI:** 10.3390/jcm12020593

**Published:** 2023-01-11

**Authors:** Kazuhiro Murakami, Tasuku Yoshimoto, Kazuhiro Hori, Rikako Sato, Ma. Therese Sta. Maria, Pinta Marito, Hinako Takano, Aye Mya Mya Khaing, Takashi Nokubi, Takahiro Ono

**Affiliations:** 1 Division of Comprehensive Prosthodontics, Faculty of Dentistry and Graduate School of Medical and Dental Sciences, Niigata University, Niigata 951-8514, Japan; 2 Department of Prosthodontics, College of Dentistry, Manila Central University, Caloocan 1400, Philippines; 3 Department of Prosthodontics, Faculty of Dentistry, Universitas Indonesia, Jakarta 10430, Indonesia; 4 Osaka University, Suita 565-0871, Japan; 5 Department of Geriatric Dentistry, Osaka Dental University, Osaka 540-0008, Japan

**Keywords:** gummy jelly, masticatory performance, oral function, mastication

## Abstract

Evaluation of masticatory ability has become more important in an aging society because decreased masticatory ability has the potential to affect the general health of older people. A new masticatory performance test, intended for older people with low masticatory ability, has been developed using gummy jelly half the size of that used in the conventional masticatory performance test. This study aimed to investigate the compatibility between the new and conventional tests and the adaptation of the new test. The new test using the 8-grade visual score with half-size gummy jelly was performed among 137 removable denture wearers (mean age 75.8 ± 9.0 years) with low masticatory performance (a score of ≤4 on a conventional test). The correlation between the scores of half-size gummy jelly (VS-H) in the new test and those of full-size gummy jelly (VS) in the conventional test was evaluated. VS-H among the groups divided by VS were also compared. A strong positive correlation was detected between VS-H and VS (*r*_s_ = 0.70). In groups with VS of 0 and 1, VS-H values were widely distributed from 0 to 7. There were significant differences in VS-H between the groups with VS of 0–2 but no significant differences in VS-H between the groups with VS of 2–4. Therefore, the masticatory performance test using half-size gummy jelly is suitable for a detailed evaluation of masticatory ability in older people with low masticatory ability when their visual score of full-size gummy jelly in the conventional test is 2 or less.

## 1. Introduction

Mastication is a very complex process in which various oral organs such as teeth, tongue, cheeks, temporomandibular joint, and masticatory muscles work together [1,2], and it is an indispensable part of nutrient intake. Aging-associated environmental changes in the oral cavity such as tooth loss [3], loss of tongue muscle strength [4], and reduced masticatory muscle mass [5] decrease masticatory ability. Decreased masticatory ability leads to the deterioration of eating habits [6] and has the potential to be one of the causes of metabolic syndrome [7], malnutrition [8], and the occurrence of aspiration pneumonia [9]. Thus, it is increasingly becoming important to quantify, objectively evaluate, and detect deterioration of masticatory ability in the super-aged society.

The comminution test is a popular method for evaluating masticatory ability, and is a method for evaluating masticatory performance based on the degree to which food crushes after biting [10]. The comminution tests have been developed in the past using various test foods such as nuts and carrots [11]. However, the type and shape of test food are very important factors when developing a comminution test that corresponds to the characteristics of older people, such as the large individual differences in oral conditions and the high percentage of people wearing dentures.

**Figure 1:**
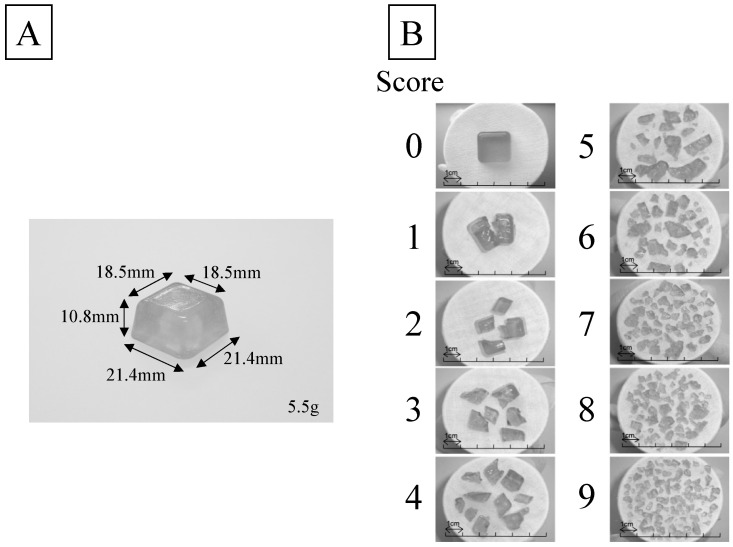
Evaluation of masticatory performance with full-size test gummy jelly. (**A**) A sample of the full-size test gummy jelly. (**B**) The visual scoring table of full-size test gummy jelly. Only patients with the visual score of 4 or less were included in this study.

So far, we have focused on the following properties of gummy jelly: (1) gummy samples are homogeneous, (2) there is no pain even if a bit fragment becomes stuck between the denture and the mucous membrane, (3) its adhesiveness is low and it does not adhere to the denture or teeth, and (4) its mechanical properties as foodstuffs can be freely adjusted. We previously developed a method for evaluating masticatory performance by measuring the increased surface area of the chewed pieces of a gummy sample after a participant had chewed it 30 times and spat it out (Figure 1) [12]. This evaluation method has been used in various situations, such as the evaluation of prosthetic treatment outcomes [13] and epidemiological studies of oral function in older people [14]. However, especially in older patients wearing complete dentures, there are many cases in which gummy samples cannot be sheared off due to a significant decrease in occlusal force [15] and masticatory muscle mass [5]. It is difficult to correctly evaluate the differences in masticatory ability among these older people, minute changes in the masticatory ability within individuals, and the effects of prosthetic treatment and oral function training. Therefore, we developed a masticatory performance test for older people with low masticatory ability [16].

This new method is based on the property that for the same food sample, smaller sizes are easier to bite. The test sample in the new method is a “half-size gummy jelly,” which contains the same material as a full-size gummy jelly that has been used in the conventional test but has only half the volume. For this method, similar to the conventional method [12], the crushing of the gummy jelly after chewing 30 times is visually evaluated as a chewing performance score from 0 to 7 using a score table. In the previous report, the validity of a score chart for the half-size gummy jelly method has been verified [16]. However, the relationship between the masticatory performance scores evaluated via this method and the conventional method is unclear. Further, the suitable cutoff score of the conventional method has not been investigated in relation to the use of the newly developed method.

Therefore, in this study, we aimed to evaluate the relationship between the masticatory performance scores obtained using the half-size gummy jelly and the full-size gummy jelly, targeting older people with low masticatory ability. In addition, we aimed to examine the adaptations to the new masticatory performance test. Here, we hypothesized that a positive correlation exists between masticatory performance scores obtained using half-size gummy jelly and full-size gummy jelly.

## 2. Materials and Methods

### 2.1. Participants

The study participants were denture wearers aged 65 years or older with a conventional masticatory performance test score of 4 or less (Figure 1) and who visited the Department of Removable Prosthodontics and General Dentistry at the Niigata University Medicine and Dental Hospital from 2015 to 2021 for dental maintenance. The exclusion criteria were oral/facial pain at the examination, severe periodontitis, severe symptom of temporomandibular disorders, jaw defect or dysphagia, neuromuscular disease, and an inability to follow the instructions of the operator due to conditions such as dementia. All study participants received an explanation of the purpose of this study in advance and only participated after providing informed consent.

Assuming that the correlation between the results of two types of masticatory performance tests was to be analyzed, the optimum sample size was determined to be 134 using G*Power (Universität Düsseldorf, Düsseldorf, Germany) with a statistical power of 0.95 and an effect size of 0.3. This study was conducted in accordance with the guidelines of the Declaration of Helsinki. The study protocol was approved by the Ethics Committee of the Faculty of Dentistry, Niigata University (approval no. 2015-3038).

### 2.2. Data Collection

The participants were seated in a reclining chair such that their Frankfurt plane was parallel to the ground, and the conventional gummy test was administered once [12]. The participants were asked to freely chew 5.5 g of full-size test gummy jelly (UHA Mikakuto, Osaka, Japan) 30 times without swallowing, and then spit it out on a piece of gauze spread out in a paper cup. Next, the piece of gauze and the saliva adhering to the chewed sample were removed with running water. Then, the degree of crushing of the sample was evaluated by the pre-trained examiner using a 10-level score table (Figure 1); this score was defined as the visual score (VS) of masticatory performance. VS of 4 was considered to correspond to the average of the bottom 25% group of older people in terms of masticatory performance [17].

**Figure 2:**
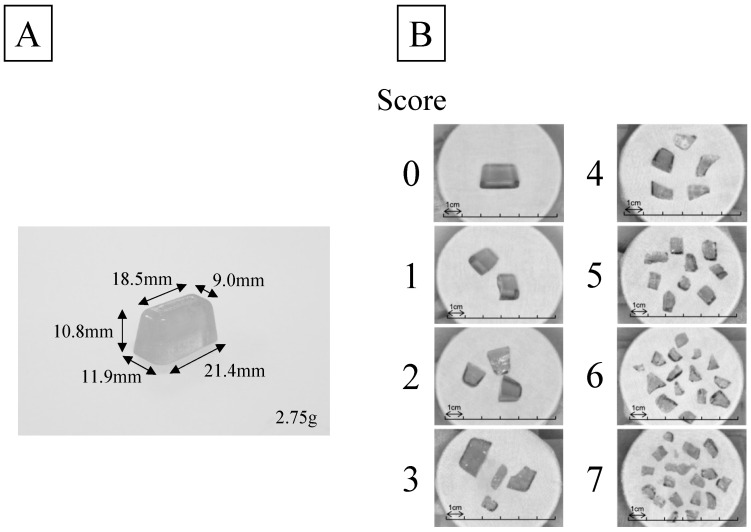
Evaluation of masticatory performance with half-size test gummy jelly. (**A**) A sample of the half-size test gummy jelly. (**B**) The visual scoring table of half-size test gummy jelly. The gummy jelly used in this new test is half the size of that used in the conventional test (Figure 1A), and the score table is different from that of the conventional test (Figure 1B).

After measuring VS, for participants with VS of 4 or less, a half-size gummy test [15] was administered once. Participants were asked to chew 2.75 g of gummy jelly (a half-size gummy jelly, UHA Mikakuto, Osaka, Japan) 30 times in the same steps as in the conventional method. Then, the degree of crushing of the sample was evaluated using an eight-level score table (Figure 2); this score was defined as the visual score by half-size gummy jelly (VS-H). A resting period of 1 min was provided between measurements, and the participants were asked to gargle several times.

The following basic information was also investigated: the participant’s age, sex, number of remaining teeth, and the Eichner index [18], a classification system for occlusal support.

### 2.3. Statistical Analysis

Spearman’s rank correlation coefficient was used to examine the relationship between VS-H and VS. In addition, participants were classified into five groups (0, 1, 2, 3, 4) according to the VS value, and the Kruskal–Wallis test was used to compare VS-H values among groups. The Mann–Whitney *U* test with Bonferroni correction was performed for multiple comparisons. The statistical analyses were performed using SPSS Statistics version 25 (IBM Japan, Tokyo, Japan) at a significance level of 5%.

## 3. Results

### 3.1. Participant Characteristics

A total of 137 participants (52 males, 85 females; average age 75.8 ± 9.0 years) were included in this study. The average number of remaining teeth among the participants was 8.3 ± 7.8. Regarding the Eichner index [18], there were 2 participants in group A (all occlusal contacts in the posterior region were preserved), 48 participants in group B (missing one or more occlusal contacts in the posterior region), and 87 participants in group C (loss of maxillary occlusal contact). Seventy-five participants (54.7%) wore complete dentures. Table 1 details the baseline characteristics of the participants.

### 3.2. Relationship between the Two Types of Gummy Jelly Regarding Masticatory Performance

**Figure 3:**
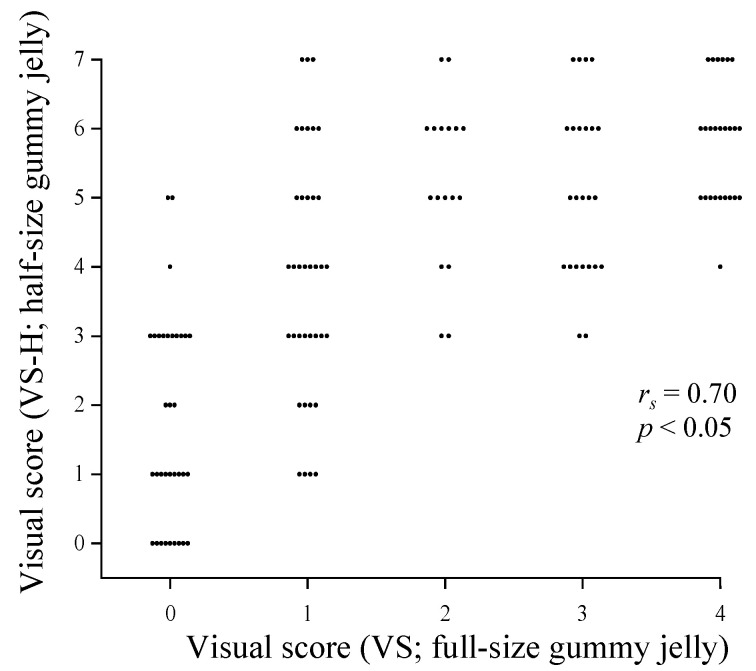
Relationship between masticatory performances determined using the two types of gummy jelly. The cluster of dots indicates the number of participants. The visual score showed a positive correlation between the test with half-size gummy jelly and that with full-size gummy jelly. Spearman’s ranked correlation coefficient (*p* < 0.05).

A strong positive correlation was observed between VS-H and VS (*r_s_* = 0.70) (Figure 3). However, among participants with VS of 0 and 1, VS-H values were widely distributed from 0 to 5 and 1 to 7, respectively.

### 3.3. Comparison of Masticatory Performances between the Half-Size Gummy Test and The Conventional Gummy Test

**Figure 4:**
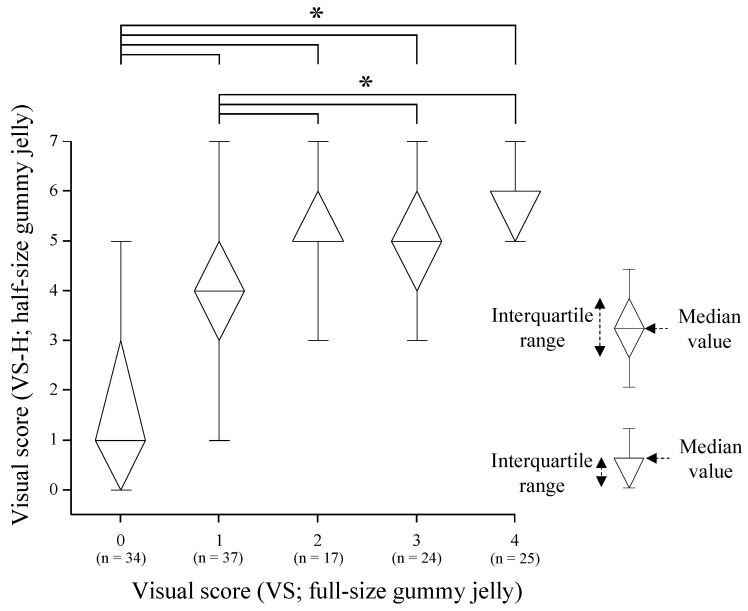
Differences in visual score (VS-H; half-size gummy jelly) among the different visual score groups (VS; full-size gummy jelly)**.** VS-H significantly increased as VS increased from 0 to 2. There were no significant differences in VS-H and VS values of 2 to 4. Kruskal–Wallis test and post hoc test with Bonferroni’s correction (*: *p* < 0.05).

Figure 4 shows the results obtained in the comparison of VS-H between groups classified by VS value. The group with a VS of 0 showed a lower VS-H value than the other groups (VS of 1 to 4) and a significant difference was observed. The group with a VS of 1 showed a lower VS-H value than the groups with VS values of 2, 3, and 4, and significant differences were observed. However, no significant differences were observed among groups with VS values of 2, 3, and 4.

## 4. Discussion

The purpose of this study was to evaluate the relationship between the masticatory performance scores obtained using half-size gummy jelly and those obtained using full-size gummy jelly. Further, we clarified the applicable range of participants for the new method of evaluating masticatory performance. Although many methods have been developed to evaluate masticatory ability [10], to the best of our knowledge, this is the first study to evaluate a method of masticatory ability assessment that focuses on participants with low masticatory ability.

The present study revealed a strong positive correlation between VS-H and VS. This was because the test samples for both tests comprised gummy foods with the same ingredients and composition. In addition, the evaluation methods of both tests were based on the same measurement principle of the degree to which the test sample was crushed. The previous study which evaluated the compatibility of two tests of masticatory ability using gummy jellies of different sizes and hardness reported that the test results showed a positive correlation, which supports the findings of the present study [19].

VS-H was widely distributed from 0 to 7 even in participants with a VS of 0 or 1, that is, participants whose test specimens could not be cut at all or could only be divided into two. Previous studies have shown that tooth loss reduces occlusal force [15] and masseter muscle mass [5] in older people. Salazar et al. also reported that the median masticatory performance among patients with new complete dentures corresponded to a VS of 1 [13]. Kosaka et al. reported that the average masticatory performance of patients in the Eichner index group C corresponded to a VS of 2 [14]. Since approximately half of the participants in this study wore complete dentures, many participants could not break the conventional gummy jelly at all. However, the cross-sectional area of the half-size gummy jelly was approximately half that of the gummy jelly used in the conventional method. Therefore, the force required to break the gummy jelly could be smaller than that of the gummy jelly used in the conventional method, making it easier to crush. This might have led to a widely distributed VS-H even in participants with a VS of 0 or 1. Therefore, VS-H has the potential for evaluating the masticatory performance of participants with a VS of 0 or 1 in detail.

On the other hand, the half-size gummy jelly was easier to crush than the full-size gummy jelly used in the conventional method; thus, it was confirmed that the score reached a plateau as the VS increased. In the present study, we divided the participants according to the VS value and compared the VS-H. Among participants with a VS of 0 to 2, VS-H increased in a stepwise manner; however, there were no significant differences in VS-H between the VS 2 group and the VS 3 and 4 groups. In other words, for participants with a VS of 3 or higher, it is possible that the VS-H will not differ due to the ceiling effect. These discussions on the ease of crushing gummy may be clarified by measuring mastication with electromyography in the future study.

Our study had several limitations. First, this study only included older adults with VS values between 0 and 4, the lower half of the VS of the conventional gummy test. To explore the range of participants suitable for the test with half-size gummy jelly, it may have been necessary not to limit the VS values among participants in this manner. However, the ceiling effect in VS-H was observed when the VS was between 2 and 4, suggesting that there was no problem with this selection criterion. In addition, in this study, we did not confirm the reproducibility and inter-rater reliability of the masticatory performance test using half-size gummy jelly. However, high reproducibility and inter-rater reliability have been confirmed for conventional masticatory performance test with full-size gummy jelly [12]. Thus, it is highly likely that the new method, which employs the same evaluation method except for the size of the test sample, would have yielded similar results.

Despite these limitations, the masticatory performance test using half-size gummy jelly has the potential to become an evaluation tool that can further stratify masticatory ability among participants with a VS of 2 or less. Previous studies have reported that decreased bolus-forming ability affects the occurrence of food aspiration and aspiration pneumonia [9,20]. Therefore, it is possible that the new method can be used as an indicator in the evaluation of aspiration risk and the selection of nursing care foods for people with dysphagia. In recent years, evidence has been accumulated not only for evaluating the deterioration of oral function but also for treatment and training aimed at maintaining and improving oral function [21,22,23]. In these treatments, the conventional masticatory performance test as well as the new method can be used to evaluate minute changes in the masticatory ability of older people with decreased masticatory function. In the future, we will evaluate changes in VS and VS-H in the treatments, including denture treatment and oral function training among older people with low masticatory ability.

## 5. Conclusions

There was a positive correlation between the masticatory performance scores obtained using the two gummy jellies. However, among participants with a masticatory performance score exceeding 2 with the conventional gummy jelly, a ceiling effect was observed in the masticatory performance score obtained using the half-size gummy jelly. The masticatory performance test using half-size gummy jelly is suitable for a detailed evaluation of the masticatory ability of participants with low masticatory ability who have a score of 2 or less in the conventional masticatory performance test.

## Figures and Tables

**Table 1:** Baseline characteristics of the participants.

Characteristics		Total (*n* = 137)
Age		
	65–74	55
	75–84	61
	≥85	21
Sex		
	Male	52
	Female	85
Number of remaining teeth		
	0	38
	1–9	44
	10–19	39
	≥20	16
Eichner index		
	Group A	2
	Group B	48
	Group C	87
Denture use		
	PD alone	62
	CD alone	43
	CD and PD	32

PD, partial denture; CD, complete denture.

## Data Availability

The data that support the findings of this study are available from the corresponding author upon reasonable request.

## References

[1] Hiiemae K., Heath M.R., Heath G., Kazazoglu E., Murray J., Sapper D., Hamblett K. (1996). Natural bites, food consistency and feeding behaviour in man. Arch. Oral Biol..

[2] Peck C.C. (2016). Biomechanics of occlusion—Implications for oral rehabilitation. J. Oral Rehabil..

[3] Ikebe K., Matsuda K., Kagawa R., Enoki K., Yoshida M., Maeda Y., Nokubi T. (2011). Association of masticatory performance with age, gender, number of teeth, occlusal force and salivary flow in Japanese older adults: Is ageing a risk factor for masticatory dysfunction?. Arch. Oral Biol..

[4] Morita K., Tsuka H., Kato K., Mori T., Nishimura R., Yoshida M., Tsuga K. (2018). Factors related to masticatory performance in healthy elderly individuals. J. Prosthodont. Res..

[5] Newton J.P., Yemm R., Abel R.W., Menhinick S. (1993). Changes in human jaw muscles with age and dental state. Gerodontology.

[6] Inomata C., Ikebe K., Kagawa R., Okubo H., Sasaki S., Okada T., Takeshita H., Tada S., Matsuda K., Kurushima Y. (2014). Significance of occlusal force for dietary fibre and vitamin intakes in independently living 70-year-old Japanese: From SONIC Study. J. Dent..

[7] Fushida S., Kosaka T., Nakai M., Kida M., Nokubi T., Kokubo Y., Watanabe M., Miyamoto Y., Ono T., Ikebe K. (2021). Lower Masticatory Performance Is a Risk for the Development of the Metabolic Syndrome: The Suita Study. Front. Cardiovasc. Med..

[8] Okada K., Enoki H., Izawa S., Iguchi A., Kuzuya M. (2010). Association between masticatory performance and anthropometric measurements and nutritional status in the elderly. Geriatr. Gerontol. Int..

[9] Hase T., Miura Y., Nakagami G., Okamoto S., Sanada H., Sugama J. (2020). Food bolus-forming ability predicts incidence of aspiration pneumonia in nursing home older adults: A prospective observational study. J. Oral Rehabil..

[10] Goncalves T., Schimmel M., van der Bilt A., Chen J., van der Glas H.W., Kohyama K., Hennequin M., Peyron M.A., Woda A., Leles C.R. (2021). Consensus on the terminologies and methodologies for masticatory assessment. J. Oral Rehabil..

[11] Bates J.F., Stafford G.D., Harrison A. (1976). Masticatory function—A review of the literature: III. Masticatory performance and efficiency. J. Oral Rehabil..

[12] Nokubi T., Yoshimuta Y., Nokubi F., Yasui S., Kusunoki C., Ono T., Maeda Y., Yokota K. (2013). Validity and reliability of a visual scoring method for masticatory ability using test gummy jelly. Gerodontology.

[13] Salazar S., Hasegawa Y., Kikuchi S., Kaneda K., Yoneda H., Nokubi T., Hori K., Ono T. (2021). The impact of a newly constructed removable denture on the objective and subjective masticatory function. J. Prosthodont. Res..

[14] Kosaka T., Ono T., Kida M., Kikui M., Yamamoto M., Yasui S., Nokubi T., Maeda Y., Kokubo Y., Watanabe M. (2016). A multifactorial model of masticatory performance: The Suita study. J. Oral Rehabil..

[15] Schimmel M., Memedi K., Parga T., Katsoulis J., Muller F. (2017). Masticatory Performance and Maximum Bite and Lip Force Depend on the Type of Prosthesis. Int. J. Prosthodont..

[16] Ono T., Yasui S., Kaneda K., Kikuchi S., Kida M., Kosaka T., Kikui M., Maeda Y., Nokubi T. (2016). Development of visual scoring method using a half size gummy jelly for evaluating masticatory performance. J. Masticat. Health Soc..

[17] Kikui M., Ono T., Kokubo Y., Kida M., Kosaka T., Yamamoto M., Nokubi T., Watanabe M., Maeda Y., Miyamoto Y. (2017). Relationship between metabolic syndrome and objective masticatory performance in a Japanese general population: The Suita study. J. Dent..

[18] Eichner K. (1990). Renewed examination of the group classification of partially edentulous arches by Eichner and application advices for studies on morbidity statistics. Stomatol. DDR.

[19] Murakami K., Hori K., Yoneda H., Sato N., Suwanarpa K., Sta Maria M.T., Marito P., Nokubi T., Ono T. (2022). Compatibility of two types of gummy jelly tests for detecting decreased masticatory function. Gerodontology.

[20] Feinberg M.J., Ekberg O. (1991). Videofluoroscopy in elderly patients with aspiration: Importance of evaluating both oral and pharyngeal stages of deglutition. AJR Am. J. Roentgenol..

[21] Matsuo K., Kito N., Ogawa K., Izumi A., Kishima M., Itoda M., Masuda Y. (2021). Improvement of oral hypofunction by a comprehensive oral and physical exercise programme including textured lunch gatherings. J. Oral Rehabil..

[22] Iyota K., Mizutani S., Kishimoto H., Oku S., Tani A., Yatsugi H., Chu T., Liu X., Kashiwazaki H. (2022). Effect of Isometric Tongue Lifting Exercise on Oral Function, Physical Function, and Body Composition in Community-Dwelling Older Individuals: A Pilot Study. Gerontology.

[23] Takano S., Yamaguchi K., Nakagawa K., Yoshimi K., Nakane A., Okumura T., Tohara H. (2021). Effect of isometric exercises on the masseter muscle in older adults with missing dentition: A randomized controlled trial. Sci. Rep..

